# 

**Published:** 1917-08-04

**Authors:** 


					Rates or Scbschiption : Twelve months, 6/6; Six months, 4/-; three months, 2/-, post free to any address in the United Kingdom.
Abroad, Twelve months, 8/8; Six months, 5/-; Three months, 2/6, post free. THE HOSPITAL "Index'' to Volume LXI.,
October 1916 to March 1917, is now ready, price 2ld. per copy, post free. Address The Manager, Thb Hospitai, " TEe
Hospital " Building, 28/29 Southampton Street, Strand, London, W.C. 2.
A NEW TREATMENT FOR GONORRHEA.'
By CHARLES RUSS, M.B., M.R.C.S.,
Physician-in-Charge, Electro-Therapeutic Department,
Male Lock Hospital, London.
Demy 8vo, 3s. net; post free, 3s. 4d?
"We have read this book with interest and profit, and cordially recom-
mend it to the profession."?The Medical Press.
" This form of treatment merits further attention, as it is both scientific
and ingenious."?British Medical Journal.
A New Edition is in preparation.
London : H. K. LEWIS & CO., Ltd., 136 Gower Street, W.C

				

